# Validation of Varian’s SmartAdapt® deformable image registration algorithm for clinical application

**DOI:** 10.1186/s13014-015-0372-1

**Published:** 2015-03-31

**Authors:** Ihab S Ramadaan, Karsten Peick, David A Hamilton, Jamie Evans, Douglas Iupati, Anna Nicholson, Lynne Greig, Robert J W Louwe

**Affiliations:** Department of Radiation Oncology, Wellington Blood and Cancer Centre, Wellington Hospital, Private Bag 7902, 6242 Wellington, New Zealand; Current address: Liz Plummer Cancer Care Centre, Cairns, Australia

**Keywords:** Deformable image registration, Intra-observer variation, Head and neck cancer

## Abstract

**Background:**

Re-contouring of structures on consecutive planning computed tomography (CT) images for patients that exhibit anatomical changes is elaborate and may negatively impact the turn-around time if this is required for many patients. This study was therefore initiated to validate the accuracy and usefulness of automatic contour propagation for head and neck cancer patients using SmartAdapt® which is the deformable image registration (DIR) application in Varian’s Eclipse™ treatment planning system.

**Methods:**

CT images of eight head and neck cancer patients with multiple planning CTs were registered using SmartAdapt®. The contoured structures of target volumes and OARs of the primary planning CT were deformed accordingly and subsequently compared with a reference structure set being either: 1) a structure set independently contoured by the treating Radiation Oncologist (RO), or 2) the DIR-generated structure set after being reviewed and modified by the RO.

**Results:**

Application of DIR offered a considerable time saving for ROs in delineation of structures on CTs that were acquired mid-treatment. Quantitative analysis showed that 84% of the volume of the DIR-generated structures overlapped with the independently re-contoured structures, while 94% of the volume overlapped with the DIR-generated structures after review by the RO. This apparent intra-observer variation was further investigated resulting in the identification of several causes. Qualitative analysis showed that 92% of the DIR-generated structures either need no or only minor modification during RO reviews.

**Conclusions:**

SmartAdapt is a powerful tool with sufficient accuracy that saves considerable time in re-contouring structures on re-CTs. However, careful review of the DIR-generated structures is mandatory, in particular in areas where tumour regression plays a role.

## Background

CT-images used for radiotherapy planning constitute only a snapshot in time of the patient’s anatomy before the start of radiotherapy. The whole treatment commonly involves 25–35 fractions, and is delivered in a time span of several weeks. During this period, the patient may undergo significant anatomical changes as a result of weight loss, tumour shrinkage or inflammation that affect the size, shape, and location of target volumes and organs at risk (OARs) [1]. Such changes can result in reduced doses to target volumes and/or increased doses to OARs [1,2] that necessitates adapting the treatment plan. Adaptive radiotherapy uses additional images to adjust the original treatment plan and counteract these anatomical changes [3-5]. However, frequent adaptation of treatment plans may substantially increase clinical workload and requires automation of several stages of treatment preparation to do this in a safe and efficient manner.

At the Wellington Blood and Cancer Centre (WBCC), on-treatment imaging using a cone beam CT (CBCT) is used to verify the reproducibility of patient positioning and assess possible changes in the anatomy of the patient. As the impact of anatomical changes and/or patient positioning strongly depends on the individual patient and the details of the treatment plan, no general rules are applied to indicate re-CTs and re-planning at the WBCC. Rather, on-treatment imaging is reviewed weekly on an individual patient basis by a multi-disciplinary team of radiation therapists, medical physicists and radiation oncologists to verify the reproducibility of patient positioning and assess anatomical changes. If this raises concern regarding target coverage or OAR sparing, a re-CT is indicated. Subsequently, the initial treatment plan is copied onto the new CT, and the dosimetric changes are assessed. If these dosimetric changes show that the target coverage or OAR sparing may be compromised, the relevant anatomical structures are re-contoured by the RO and a new treatment plan is created. Re-contouring of all relevant structures is time consuming and limits the number of patients for whom plan adaptation can be achieved in a short turn-around time. Deformable Image Registration (DIR) may be useful to quantify the detected anatomical changes and to automate re-contouring of anatomical structures after an anatomical change has been observed. Clinical implementation of DIR has been validated by several groups [6-8]. However, these studies did not include all aspects that were relevant to DIR implementation at the WBCC. For instance, none of these studies specifically addressed the accuracy of DIR when applied to a contrast-enhanced planning CT and a non-contrast enhanced repeat CT, or investigated the time gained for ROs to review and modify DIR-generated structures instead of re-contouring structures from ‘scratch’. Furthermore, Hardcastle *et al*. [6] investigated two DIR algorithms that are available in Pinnacle (Philips Medical Systems, Andover, MA), but are different from the ‘accelerated demons’ algorithm available to Varian users. The clinical implementation of the latter algorithm was validated by Wang *et al*. [7] using the original, in-house developed version of the ‘accelerated demons’ algorithm by the same group [9]. It seems highly likely that additional modifications were made to this original source code for the commercial implementation in Varian’s SmartAdapt®. For instance, the previous version of SmartAdapt (v.10) offered the option to either include smoothing in the DIR process or not, while the current version (v.11) as used at the WBCC only offers the ‘standard DIR algorithm’ and has fixed parameter settings. Finally, Eiland *et al*. [8] did use the same version of SmartAdapt as currently available at the WBCC, but used a different approach involving the DIR of the planning CT to an on-treatment Cone Beam CT, which is not applicable for the workflow investigated in this study. These observations warranted an independent validation of the DIR implementation in Varian’s SmartAdapt® at the WBCC. Therefore, this retrospective study was started to validate the clinical implementation of DIR, and it specifically reports on:

1. The DIR accuracy in adapting structures using Varian’s SmartAdapt®;
2. Differences between delineated structures either modified after DIR by an RO or contoured from ‘scratch’ as per current protocol;
3. The DIR accuracy when registering a contrast-enhanced planning CT and a non-contrast enhanced repeat CT;
4. Quantitative and qualitative analysis of the results obtained for the gross target volume (GTV), clinical target volumes (CTVs) for all dose prescription levels as well as multiple OARs: brain stem, spinal cord and both parotid glands;
5. Time saving compared to the current clinical workflow at the WBCC.

## Methods and materials

### Patient selection

This study was carried out retrospectively using the data of eight head and neck cancer patients treated at the WBCC treated by four ROs (Table 1). Multiple planning CTs were available for the selected patients, either because anatomical changes were observed during treatment or because the CT position could not be reproduced during treatment with sufficient accuracy to warrant safe treatment. All patient plans and image sets selected were anonymized, and clinically contoured structures were copied to a non-clinical test database. For each patient, the first CT scan was acquired with iodine-based CT contrast administered but subsequent CTs were acquired without CT contrast. The slice thickness for all CTs was 3 mm. For two patients (P2 and P7), a third CT scan acquired during the course of treatment was available.

**Table 1:** **Clinical information for the 8 head and neck cancer patients included in this study**

	**P1**	**P2**	**P3**	**P4**	**P5**	**P6**	**P7**	**P8**
**Tumour Site**	L Tonsil	L Tonsil	R Tonsil	Nasopharynx	L Tongue	R Tonsil	Para-nasal sinus	Tongue
**No. fractions**	30	30	30	30	30	30	30	30
**Surgery**	Yes	No	No	No	Yes	No	Yes	No
**Staging**	T2N1M0	T3N2bM0	T1N0M0	T1N0M0	T2N2bM0	T2N2M0	T4N2M0	T4N3aM0
**GTV [** **cm** ^3^ **]**	14.1^a^	88.8	26.2	82.8	-	47.5	-	201.1
**CTV**_50–54_ **[cm** ^3^ **]**	201.8	446.1	144.6	567.5	299.4	314.1	-	179.1^b^
**CTV**_60_ **[cm** ^3^ **]**	-	-	-	-	214.3	-	583.4	331.7
**CTV**_66_ **[cm** ^3^ **]**	68.5	186.8	38.8	153.2	57.7	108.1	-	-
**Additional imaging**	PET	PET	-	MRI	MRI	-	MRI	-
**Number of CTs**	2	3	2	2	2	2	3	2
**Days after CT**_1_	42	26/42	36	30	25	35	42/69	31
**Days after RT start**	25	8/24	21	16	7	-^c^	-^c^/20	20
**Weight change between CTs** **[kg]**	−7.1	−10.7/+0.8	−1.3	−3.8	−2.6	0.0	+1.6/- 0.8	−6.8
**Reason for re-** **CT**	Tumour shrinkage	Patient position	Swelling	Patient position	Swelling	Patient position	Patient position	Tissue Loss
**New Mask**	Yes	Yes/No	Yes	No	No	No	No/No	No
**New plan**	Yes	Yes/Yes	Yes	No	No	No	NA/No	No

^a^This GTV represents a node that was not removed during surgery.

^b^The low dose CTV for this patient was adjacent to and did not include the high dose CTV as usual.

^c^Initial start of RT was delayed due to clinical reasons. Second CT was acquired prior to actual start of RT.

### Structure selection

A number of structures as contoured by the RO during the treatment preparation phase were common in most data sets and therefore selected for analysis in this study: GTV, CTVs, spinal cord, parotid glands, and the brainstem. PTVs were not included in the analysis as these structures would not be included in the DIR process in a clinical setting but would be re-created by expanding the adapted CTV after DIR. In addition to the selected structures, the C4 vertebra which is not expected to deform in between consecutive CT scans was selected as a reference structure to verify the accuracy of the DIR process. This structure was delineated by the first author.

### Workflow

CT sets of each patient were first rigidly registered using bony anatomy as a starting point before further registration using DIR. In the latter step, the structures of interest linked to the first CT image were deformed as defined by the deformation vector field resulting from the DIR process. In order to investigate the feasibility of the intended clinical workflow, the DIR-generated contours were reviewed and if necessary modified by the treating ROs. However, these RO-modified contours could potentially include a bias towards pre-defined structures when testing the accuracy of the DIR algorithm. Therefore, another test was conducted in addition where each RO independently re-contoured the structures on the re-CT image using his/her clinical notes as well as the original structure set, but without reference to the DIR-generated structures. The DIR generated structures for the third CT scans acquired for patients P2 and P7, were obtained using the second CT scan and corresponding clinical structures as the reference data set. In addition to determining the accuracy of the DIR algorithm for clinical application, the results of the two tests were compared to investigate a potential bias when the structures resulting from DIR are only reviewed and adapted prior to clinical application, instead of completely re-contouring all structures when a new CT has been acquired. For that purpose, all data was analysed both quantitatively and qualitatively as described below.

### Quantitative analysis

For quantitative analysis, the RO-drawn contours and the DIR-generated contours were first converted into high resolution structures as they are available in Eclipse v.11 to improve the accuracy of the next analysis step. This improves the in-plane resolution of the structures but not the resolution in the longitudinal direction which is determined by the 3 mm CT-slice thickness. The next step comprised a Boolean ‘AND’- operation in the contouring module of Eclipse to determine the volumetric overlap between the RO-drawn contours and the DIR-generated contours. Subsequently, the obtained volume data were used to calculate the Dice Similarity Index (DSI) [10], which in this study represents the percentage volume overlap of two presentations A and A’ of a structure:

1 $$ DS{I}_{AA\hbox{'}}=\frac{V_A\cap {V}_{A\hbox{'}}}{\left({V}_A+{V}_{A\hbox{'}}\right)/2} $$

where *V* represents the volume of a structure. Statistical tests were carried out using Matlab (The Mathworks, Natick, MA, USA).

### Qualitative analysis

For qualitative analysis, the differences between RO-drawn and DIR-generated structures were reviewed together with the treating RO. Discrepancies between the two structures were investigated in consultation with clinical notes and the agreement between the two contours was assessed using a pre-defined scoring system based on a scale of 1–4:

1. The DIR-generated contour is more accurate than the RO-drawn contour
2. The differences between the DIR-generated contour and RO-drawn contour are minimal when clinically acceptable without modification
3. The DIR-generated contour can be accepted after minor modification
4. The DIR-generated contour requires major modification and is therefore unusable.

This scoring system is equal to the system used by Hardcastle *et al*. [6] except that the above scoring system has the first category additional to it. This category was specifically added to this scoring system considering that intra-observer variation is an important factor in head-and-neck radiation oncology and could in principle yield such scores. For instance, the DIR may produce a more consistent result based on the initial RO contours and the change in shape revealed by the change in voxel intensities in the two CT images.

### Time saving

During a pilot study that included the first five tests of this study, the RO was asked to retrospectively estimate the time required to edit the contours, effectively within a range of 10 minutes. However for all subsequent tests, the time required to either edit the contours or to delineate the structures *de novo* by the ROs was actually recorded to improve the accuracy of the estimated possible time saving after clinical implementation of SmartAdapt.

### Deformable image registration software and algorithm

The DIR software programme used in this study was the SmartAdapt® module of the Eclipse™ v.11 Treatment Planning System (TPS) from Varian (Varian Medical Systems Inc., Palo Alto, CA). This application employs a Modified Demons-based DIR algorithm [9,11]. Modification of algorithm parameters or boundary conditions that can potentially affect the DIR results could not be investigated as these are fixed in the implementation of DIR in this version of SmartAdapt.

## Results

### Accuracy of DIR generated structures

The DSI scores of the overlap between the DIR generated structures and those independently re-contoured by an RO for the 2nd CT scan of P1-P8 were on average 0.82 ± 0.08 (1 S.D.) and had a range of 0.54-0.96 (Table 2). All DSI scores appeared to be lower than expected, in particular the DSI scores for the OARs for most patients. In addition, the average DSI scores seem to vary from patient to patient, with very low DSI scores for P8 in particular for whom the CT images did display a poor soft-tissue contrast. A Kruskal-Wallis test including a post-hoc test using a Bonferroni correction for multiple comparisons [12,13] showed that the difference between the average DSI score for patient P8 was significantly lower than those obtained for patients P1-P7 (p < 0.01). Similarly, it could be shown that the average DSI score for the spinal cord was significantly lower than those for other structures (p < 0.01), while the average DSI score for the right parotid was not significantly lower (p = 0.18).

**Table 2:** **DSI scores reflecting the overlap between the DIR-generated and RO re-contoured structures on a repeat CT-scan**

	**P1**	**P2**	**P3**	**P4**	**P5**	**P6**	**P7**	**P8**	**Mean**	**S.D**
**GTV**	0.80	0.83	0.84	0.89		0.93		0.77	**0.84**	**0.06**
**CTV**_54_	0.88	0.89	0.84	0.87	0.91	0.92		0.84	**0.88**	**0.03**
**CTV**_60_					0.91		0.88	0.86	**0.89**	**0.02**
**CTV**_66_	0.85	0.92	0.82	0.89	0.87	0.94			**0.88**	**0.04**
**Brain Stem**	0.83	0.96	0.67	0.92	0.88	0.91	0.80	0.68	**0.83**	**0.11**
**Spinal Cord**	0.78	0.84	0.73	0.75	0.74	0.78	0.77	0.63	**0.75**	**0.06**
**R Parotid**	0.73	0.76	0.79	0.74	0.90	0.90	0.77	0.54	**0.77**	**0.11**
**L Parotid**	0.82	0.77	0.84	0.86	0.82	0.91	0.82	0.71	**0.82**	**0.06**
**Mean**	**0.81**	**0.85**	**0.79**	**0.84**	**0.86**	**0.90**	**0.81**	**0.72**		
**S.D**.	**0.05**	**0.07**	**0.07**	**0.07**	**0.06**	**0.05**	**0.05**	**0.12**		

CTV_x_ denotes the clinical target volume with a dose prescription of x Gy.

### Analysis of a potential bias

**Figure 1:**
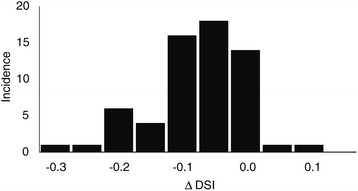
**Histogram of the differences between DSI scores for RO re**-**contoured and RO**-**modified structures.** The bars include the data of all structures of patients P1-P8 as displayed in Tables 1 and 2.

On average the DSI scores of the overlap between DIR generated and RO-modified structures were 0.93 ± 0.08 (1 S.D.) (Table 3). A histogram of the differences between the individual DSI scores of the two tests showed that the DSI scores reflecting the overlap of the DIR-generated and RO re-contoured structures are lower than those reflecting the overlap of the DIR generated and RO-modified structures in nearly all cases (Figure 1). A paired two-sided Wilcoxon sign rank test confirmed that this difference was statistically significant (p < 0.001). This difference may reflect intra-observer variation, but the significantly higher DSI scores close to unity for the RO-modified structures also indicate that there is probably a bias towards approving the DIR generated structures and possibly only a limited number of changes would be made in a clinical setting. This apparent bias also reduced the differences in DSI scores between specific patients and structures that were observed previously for the overlap between DIR-generated and RO-drawn contours. For the overlap of RO-modified structures and DIR-generated structures, none of the average DSI scores of specific patients and structures were significantly different (p > 0.05).

**Table 3:** **DSI scores reflecting the overlap between the DIR-generated and RO-modified structures on a repeat CT-scan**

	**P1**	**P2**	**P3**	**P4**	**P5**	**P6**	**P7**	**P8**	**Mean**	**S.D**
GTV	0.90	0.83	0.99	0.97		0.98		0.97	**0.94**	**0.06**
CTV54	0.95	0.97	0.99	0.98	0.99	0.99		0.96	**0.98**	**0.01**
CTV60					0.99		0.94	0.97	**0.96**	**0.03**
CTV66	0.97	0.87	0.95	0.98	0.98	0.98			**0.95**	**0.04**
Brain Stem	0.86	1.00	0.68	0.98	0.98	0.95	0.87	0.65	**0.87**	**0.14**
Spinal Cord	1.00	0.95	0.82	0.89	0.89	0.91	0.77	0.91	**0.89**	**0.07**
R Parotid	0.89	1.00	1.00	0.98	0.98	0.95	0.90	0.95	**0.95**	**0.04**
L Parotid	0.93	1.00	0.88	0.98	0.97	0.94	0.85	0.74	**0.91**	**0.09**
**Mean**	**0.93**	**0.94**	**0.90**	**0.97**	**0.97**	**0.96**	**0.86**	**0.88**		
**S.D**.	**0.05**	**0.07**	**0.12**	**0.04**	**0.04**	**0.03**	**0.06**	**0.13**		

CTV_x_ denotes the clinical target volume with a dose prescription of x Gy.

### Impact of registering contrast-enhanced and non-enhanced CT scans

Three CT-scans and corresponding structures were available for patients P2 and P7, where only the original planning CT was contrast-enhanced and both re-CTs were registered to the most recent CT-scan. The DSI scores observed for P2 and P7, for a pair of CT images with only one contrast-enhanced CT image on one hand, and two non-enhanced CT-images on the other hand, did not show a systematic difference in DIR performance (Table 4). However, the comparison above could only be made for two patients. Therefore, no conclusion could be made concerning a possible contribution of registering non-enhanced re-CTs and contrast-enhanced initial planning CT images to the relatively low DSI scores in Table 2.

**Table 4:** **DSI scores reflecting the overlap between DIR-generated and RO re-contoured structures for CT2 and CT3**

	**P2**		**P7**	
	**CT2**	**CT3**	**CT2**	**CT3**
**GTV**	0.83	0.66		
**CTV**_54_	0.89	0.87		
**CTV**_60_			0.88	0.89
**CTV**_66_	0.92	0.79		
**Brain Stem**	0.96	0.84	0.80	0.82
**Spinal Cord**	0.84	0.75	0.77	0.79
**R Parotid**	0.76	0.82	0.77	0.74
**L Parotid**	0.77	0.83	0.82	0.81
**Mean**	**0.85**	**0.79**	**0.81**	**0.81**
**S.D**.	**0.07**	**0.07**	**0.05**	**0.05**

CTV_x_ denotes the clinical target volume with a dose prescription of x Gy.

### Qualitative analysis

**Figure 2:**
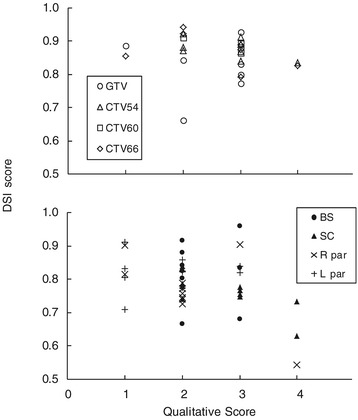
**Relation between quantitative and qualitative analysis results.** Solid markers: DSI scores reflecting the overlap between DIR-generated structures and the RO re-contoured structures as a function of the qualitative assessment score upon review by the RO.

The qualitative scores given by the ROs upon review of the DIR-generated structures and comparison with the re-contoured stuctures, showed that only 5 (8%) of the DIR-generated structures required major modifications and were not usable. Furthermore, 8 (12%), 29 (44%), and 24 (36%) of these structures were more accurate than the RO re-contoured structures, could be accepted without modifications, or required at most minor modifications, respectively. These scores showed only a weak correlation with the corresponding DSI scores (Figure 2), although DIR-generated structures that were deemed to require major modification (score 4) during qualitative review did exhibit a lower DSI score on average. For 6 delineated parotids, one GTV and one CTV, the DIR-generated structures were considered to be more accurate than the RO re-contoured structures (score 1) upon retrospective review by the RO. It was estimated that for these cases, limitations in soft-tissue contrast of the CT images resulted in intra-observer variation reflected by small differences in delineation.

### Time saving

The average time required to either re-contour, or to review and modify DIR-generated structures on the 2nd and 3rd CT images was 67 ± 30 (1 S.D.) and 29 ± 8 (1 S.D.) minutes, respectively (Table 5). In one case (P6), re-contouring of the relevant structures was slightly faster than adapting the DIR-generated structures. However, time saving for the ROs was observed for all other cases, with the average time difference equal to 38 ± 25 (1 S.D.) minutes. A paired t-test showed that the difference in required time was significant (p < 0.001).

**Table 5:** **Time required by the ROs in minutes to adapt DIR-generated structures or re-contoured structures from scratch**

**Patient**	**CT set**	**Adapt**	**Re**-**contour**	**Gain**
P1	CT2	30 (±10)	54(±1)	24
P2	CT2	30(±10)	86(±1)	56
P2	CT3	30(±10)	90(±1)	60
P3	CT2	30(±10)	89(±1)	59
P4	CT2	30(±10)	72(±1)	42
P5	CT2	30(±10)	50(±1)	20
P6	CT2	27(±1)	20(±1)	−7
P7	CT2	21(±1)	45(±1)	24
P7	CT3	15(±1)	44(±1)	29
P8	CT2	45(±1)	120(±1)	75
	**Mean**	**29**	**67**	**38**
	**S.D**.	**8**	**30**	**25**

The values between brackets represent the maximum range of the estimated/measured time (see text).

## Discussion

A commercially available DIR package was evaluated at the WBCC to verify its reliability and usefulness to generate an updated structure set for re-CT images that are occasionally acquired to verify the validity of a treatment of a patient. This evaluation revealed that only minor volumetric changes are made to DIR-generated structures upon review by an RO. Quantitative analysis showed that on average 93% of the volume of the RO-modified structures overlap with the DIR-generated structures. However, considerably smaller volumetric overlap (82%) was observed between the DIR-generated structures with those that were independently re-contoured by an RO. This highlights that there is a potential bias towards approving DIR-generated structures in a clinical setting. However, it should be noted that this bias is likely also accompanied by a decrease in intra-observer variation after implementation of DIR (see further below).

### Differences between DSI scores of structures or patients

The average DSI scores for the overlap between DIR-generated and RO-drawn contours were found to be significantly different for patient P8 and for the spinal cord. These differences may have been related to poor soft-tissue contrast that was specifically noted for P8 and is not uncommon for parts of the spinal cord. However, the average DSI scores were not significantly different for the parotids where it may have been expected as well. It should be noted that with multiple ROs, each delineating the structures of a single patient, and the variation of structures between the various patients, the data set is relatively inhomogeneuous, and the results of statistical testing should be interpreted with caution. Furthermore, the absence of significant differences for specific patients or structures when comparing the average DSI scores of the overlap between DIR-generated and RO-modified contours is likely caused by the bias towards approving DIR-generated contours.

### Differences between quantitative and qualitative results

Qualitative analysis of the results using a pre-defined scoring system at the review of the DIR-generated structures by an RO showed only a weak correlation with the DSI scores calculated during quantitative analysis (Figure 2). However, the two analysis methods are fundamentally different and are complementary in a number of aspects. For instance, contour changes that are scored as minor changes during qualitative analysis do not always represent the same change in volumetric overlap. Furthermore, qualitative analysis and scoring include the clinical relevance of discrepancies and are accordingly prioritised as judged by an RO. In a clinical setting, these considerations will be fundamental in approving DIR-generated structures while small inaccuracies, for instance in delineation of the spinal cord at locations well away from the irradiated volume, will generally be dismissed in a clinical setting. Therefore, the results of the qualitative analysis based on the review of results by the ROs were considered to be pivotal in the assessment of the accuracy and usefulness of the DIR package in this study. This qualitative analysis showed that the majority of DIR-generated structures could be approved after some modification, and that DIR resulted in a considerable time saving. The role of the quantitative analysis was to highlight potential failure modes such as bias towards approving DIR-generated structures, inconsistencies in contouring, and intra-observer variability.

### Review of discrepancies

**Figure 3:**
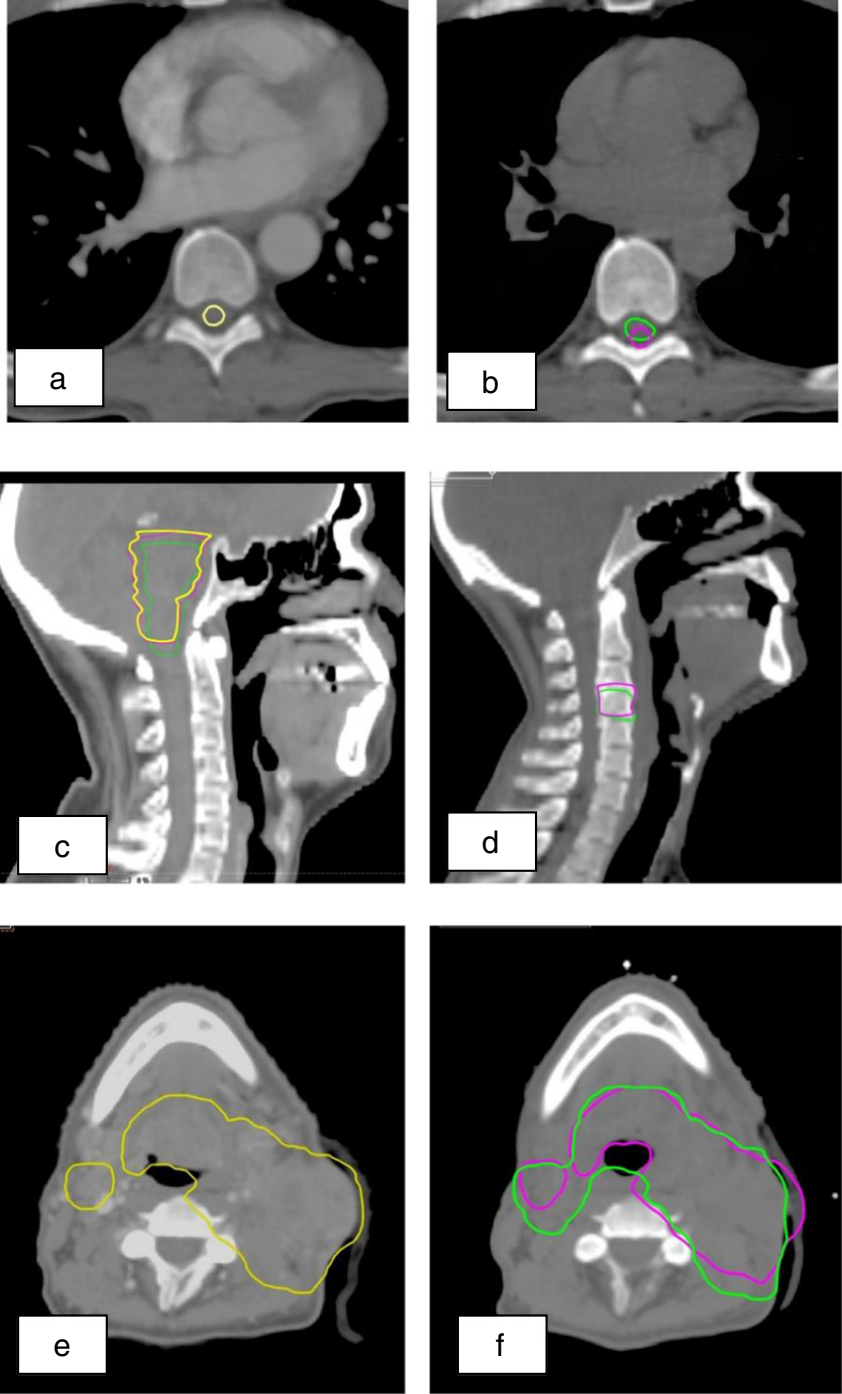
**Comparison of DIR**-**generated** **(purple),** **RO re-** **contoured** **(green),** **and initially delineated planning CT structures** **(yellow).** Panels **(a/b)** show an example where the low soft-tissue contrast induced an incorrect shift of DIR-generated structure of the spinal cord; **(c)** intra-observer variation in the delineation of the brain stem; **(d)** uncertainty at the cranial and caudal boundary of C4 delineation due to limited slice resolution; **(e/f)** difference between DIR-generated and the RO-modified CTV after tumour regression.

Further inspection of overlap between DIR generated and RO re-contoured structures revealed a number of common causes for discrepancies:

- *Limitations in soft-tissue contrast in CT images* Limitations in soft-tissue contrast in CT imaging hamper accurate delineation of specific organs such as parotids, spinal cord and brain stem. In addition, the distinction between individual vertebrae that are compressed, or between the external patient contour and build-up material, can be difficult. As the DIR algorithm uses the differences in Hounsfield units (HUs) to track patient deformation, these limitations may result in a lower DSI score. Figure 3a,b displays an example of this limitation resulting in a low DSI score for the spinal cord.
- *Intra-observer variation* For a number of delineated structures, considerable differences were observed between the delineation of the same structure on the two consecutive CTs. Inspection of the scan acquisition settings revealed a variation in mAs settings but this did not noticeably affect the soft-tissue contrast of the CT images in this study. Although limitations in soft-tissue contrast may be an important cause of intra-observer variation, some of the observed differences clearly reflected a difference in interpretation of the two CT images that could not be explained by limitations in soft-tissue contrast only. Furthermore, some discrepancies were caused by the availability of new and/or additional information in clinical notes. Figure 3c shows an example of intra-observer variation in delineation of the brainstem.
- *Differences in CT-contrast enhancement* Although no systematic difference between the DSI scores for data sets with or without contrast enhanced CT images could be demonstrated, it was estimated that contrast enhancement did impact the accuracy of the DIR result in a number of cases where structures were proximal to relatively large blood vessels.
- *Errors due to a limited CT slice resolution* The CT slice resolution applied for treatment preparation in this study was 3 mm which is the default CT slice resolution for the majority of patients at the WBCC. Any uncertainty in the actual deformation at the superior and inferior ends of delineated structures can therefore potentially result in a 3 mm error at these locations and a decrease in overlap of DIR-generated and RO re-contoured structures. Figure 3d displays an example of this limitation resulting in a low DSI score for C4.
- *Clinical target volume not adapted for tumour shrinkage during treatment* Tumour shrinkage in between the two consecutive CT images was observed for a number of patients. In those instances the DIR algorithm seemed to be able to closely track this phenomenon. However, as no proof exists that all microscopic lesions have been sterilised, changes to the CTV during the course of treatment are usually only applied very reluctantly. This was observed for a number of cases and contributed to a lower DSI score (Figure 3e-f).

### Observer variability

Intra and inter-observer variability in structure delineation in head-and-neck radiotherapy has been described by numerous authors [14-17]. Similarly, in this study several examples of intra-observer variability could be identified. Review of the discrepancies between the structure sets by an RO and a physicist revealed a number of common causes for these differences including poor image contrast, and the impact of a limited CT slice resolution. In addition, re-contouring clinical target volumes on a re-CT acquired mid-treatment where tumour regression or other changes in anatomy may be observed is subject to interpretation based on limited information and therefore prone to considerable observer variation [6,18]. Nevertheless, it is obviously important to reduce observer variation in delineation as much as possible and several methods have been proposed in literature including review boards, multi-modality imaging, contouring atlases and protocols [15,19,20]. Also the application of DIR imposes consistency in contouring between consecutive CT images as it uses prior contouring as starting point [14]. However, as the accuracy of the delineation of structures on the first planning CT is prone to observer variability as well, the DIR-generated structures must always be carefully reviewed by the treating RO.

### Comparison with other studies

Very similar DSI scores were found by a number of authors compared to those reported in this study. Chao *et al*. studied the reduction in delineation variation using DIR and found average DSI scores equal to 0.61 and 0.57 for the overlap between DIR-generated and independently drawn contours by ROs for base-of-tongue and nasopharyngeal tumours, respectively [14]. When the DIR-generated structures were only adapted by ROs, these DSI-scores improved to 0.90 and 0.88, respectively. Wang *et al*. found an improvement in average DSI-scores from 0.83 to 0.97 in a performance validation study of DIR [7]. Hardcastle *et al*. performed a multi-institutional evaluation of DIR algorithms and found average DSI scores of approximately 0.80 [6]. That study also applied a very similar qualitative analysis as the current study, reporting that 94% of the DIR-generated structures were usable but 6% of these required major modification, as compared to 92% and 8% in our study. Finally, Brouwer *et al*. reported concordance indices ranging between 0.64 – 0.71 for the overlap between DIR-generated structures and those independently contoured by several ROs for OARs excluding the thyroid cartilage [17]. As the concordance index is comparable with the DSI but tends to be lower for disjoint volumes, we estimate that these results are also very comparable to the results presented in this study.

## Conclusions

We have shown that the SmartAdapt is a very useful and time-saving tool for DIR application to propagate the structures delineated on the primary planning CT to consecutive re-CTs. DIR provided reasonably accurate structures in 92% of the cases that required no or only minor modifications. However, careful review of the DIR-generated structures by the treating RO remains crucial, in particular where tumour shrinkage plays a role. Furthermore, this study highlighted the presence of considerable intra-observer variation similar to findings reported in literature previously.

## Abbreviations

CBCT: Cone beam computed tomography
CT: Computed tomography
CTV: Clinical target volume
DIR: Deformable image registration
DSI: Dice similarity index
GTV: Gross target volume
OAR: Organ at risk
HUs: Hounsfield units
RO: Radiation Oncologist
TPS: Treatment Planning System
WBCC: Wellington Blood and Cancer Centre
